# Investigation on the Integration of Low-Cost NIR Spectrometers in Mill Flour Industries for Protein, Moisture and Ash Content Estimation

**DOI:** 10.3390/s23208476

**Published:** 2023-10-15

**Authors:** Vasileios Boglou, Dimosthenis Verginadis, Athanasios Karlis

**Affiliations:** Department of Electrical and Computer Engineering, Democritus University of Thrace (DUTh), 67100 Xanthi, Greece; vboglou@ee.duth.gr (V.B.); dvergina@ee.duth.gr (D.V.)

**Keywords:** nondestructive instrumentation, near-infrared spectrometry, parameter estimation, optimization algorithm, fuzzy-cognitive-maps

## Abstract

The flour milling industry—a vital component of global food production—is undergoing a transformative phase driven by the integration of smart devices and advanced technologies. This transition promises improved efficiency, quality and sustainability in flour production. The accurate estimation of protein, moisture and ash content in wheat grains and flour is of paramount importance due to their direct impact on product quality and compliance with industry standards. This paper explores the application of Near-Infrared (NIR) spectroscopy as a non-destructive, efficient and cost-effective method for measuring the aforementioned essential parameters in wheat and flour by investigating the effectiveness of a low-cost handle NIR spectrometer. Furthermore, a novel approach using Fuzzy Cognitive Maps (FCMs) is proposed to estimate the protein, moisture and ash content in grain seeds and flour, marking the first known application of FCMs in this context. Our study includes an experimental setup that assesses different types of wheat seeds and flour samples and evaluates three NIR pre-processing techniques to enhance the parameter estimation accuracy. The results indicate that low-cost NIR equipment can contribute to the estimation of the studied parameters.

## 1. Introduction

The milling flour industry—a cornerstone of global food production—is currently navigating a transformative journey driven through the integration of smart devices and advanced technologies. While these innovations hold great promise for enhancing efficiency, quality and sustainability in flour production, they also bring forth a unique set of challenges. As mills increasingly adopt smart devices, ranging from Internet of Things (IoT) sensors and automated machinery to data analytics and Artificial Intelligence (AI)-driven systems, they must confront issues such as cybersecurity threats, data management complexities, workforce upskilling and the need to strike a delicate balance between tradition and modernity. This convergence of age-old techniques and cutting-edge technology poses both exciting opportunities and formidable obstacles that require thoughtful consideration and strategic solutions [1,2].

In the flour milling industry, the accurate estimation of the protein and moisture content in wheat grains and flour holds paramount importance [3]. Wheat is a fundamental staple crop that serves as the primary source of nutrition for a substantial portion of the global population. It is a versatile grain used in various food products, ranging from bread and pasta to cereals and snacks. The quality of these end products is directly influenced by the protein and moisture content present in the wheat grains [4].

Protein content is a critical factor as it directly impacts the functional and nutritional properties of wheat-based products. Proteins, particularly gluten, play a pivotal role in determining the dough’s elasticity and strength during the baking processes. This directly affects the final product’s texture, volume and overall quality. Furthermore, the protein content also influences the nutritional value of the products, as proteins are a primary source of amino acids, which are essential for human health. Moisture content, on the other hand, is a key indicator of wheat grain quality and storability. Proper moisture levels are crucial to prevent the growth of molds, bacteria and other microorganisms that can lead to spoilage and contamination. Additionally, the moisture content affects the weight of the grains, which is a crucial factor in pricing and trading commodities. Incorrect moisture levels can lead to economic losses due to decreased product value, increased energy consumption during processing and potential storage issues [5].

The accurate estimation of the protein and moisture content is not only important for maintaining product quality, but also for complying with industry standards and regulations. Variability in these parameters can occur due to factors such as different wheat varieties, growing conditions, harvesting methods and storage conditions. Therefore, modern wheat mill industries employ advanced techniques and technologies to precisely measure the protein and moisture content. These may include Near-Infrared (NIR) spectroscopy, moisture meters and laboratory analyses.

NIR spectroscopy is a technology that uses the near-infrared region of the electromagnetic spectrum (780–2500 nm) to measure the chemical composition of materials [6]. It has been used for many years in the food industry, including the wheat milling industry, to estimate the protein and moisture content of wheat. NIR spectroscopy had become a reliable and accurate method for estimating the protein and moisture content of wheat and flour [7]. It is a non-destructive method; this makes it a valuable tool for the wheat milling industry as it allows the protein and moisture content of wheat to be measured without affecting its quality, while at the same time, there is no need to destroy any samples. Furthermore, it is a fast and efficient method. A single sample can be measured in a matter of seconds, making it ideal for in-line measurements. This means that the protein and moisture content of wheat can be measured as it is being processed, which can help to improve the efficiency of the milling process.

However, establishing state-of-the-art NIR technologies in mills requires significant investment [8]. The initial cost of implementing NIR technologies is a concern for some mill operators; however, the market has responded with low-cost NIR solutions that make this advanced technology more accessible. Affordable and compact NIR devices, such as handheld and portable analyzers, have emerged as feasible solutions for smaller mills or those with budget constraints. These low-cost NIRs offer the advantage of quick measurements, reduced dependence on lab testing and ultimately contribute to optimizing the production processes and ensuring consistent product quality [8,9].

In the context of utilizing NIR spectroscopy as a method for quantifying protein, moisture and ash levels in cereals, various computational models have been developed, with regression methods being the most prevalent choice in the literature. These regression models serve to calibrate the systems capable of providing precise parameter estimates. Within this framework, diverse regression models have been explored to establish potential correlations between protein and moisture concentrations in cereals and flours and the percentage of electromagnetic radiation absorption at specific wavelengths. In essence, regression models facilitate the quantification of the relationship between two parameters [10]. Among the models applied are Single Linear Regression (SLR) [11], Least Squares Support Vector Machine Regression (LSSVR) [12], Partial Least Squares (PLS) [13] and Neural Networks (NN) [14]. Particularly noteworthy, the LSSVR model has demonstrated its effectiveness in calibrating and estimating protein levels in cereals and flours, exhibiting robustness and achieving a correlation coefficient exceeding 98% [15]. In the context of employing the LSSVR model to calibrate systems for protein concentration estimation in food products, it was observed that specific wavelengths in the near-infrared spectrum—namely 1178, 1382, 1498, 1670, 1768, 1888, 1970, 2064, 2146, 2278, 2302, 2444 and 2490 nm—were sensitive to this parameter [15].

In this study, we investigate the potential of a compact, portable NIR scanner for assessing the protein, moisture and ash content in grain seeds and flour by installing an experimental setup in a mill flour industry. To facilitate this examination, we propose a novel estimation model based on the application of fuzzy cognitive maps theory (FCMs). One of the key advantages of FCMs is their ability to capture and model complex, uncertain and interdependent relationships in various domains, making them valuable for decision support and systems analysis. To the best of our knowledge, this is the first effort of utilizing FCMs in such applications. We establish an experimental setup for evaluating various types of wheat seeds and flour. Three different NIR pre-processing techniques are examined in a set of wheat and flour samples to investigate their effectiveness in the estimation of the required parameters. The findings suggest that the integration of a miniature NIR scanner coupled with parameter estimation models based on FCMs and employing computational intelligence techniques offers a cost-effective and viable alternative to achieve accurate results. Finally, the proposed FCM models are compared to the well-established partial least squares regression (PLS) method to evaluate its effectiveness in regard to the studied problem. The results indicate that the proposed FCM-based models can improve the accuracy of the parameters estimation.

This paper is structured as follows: The Section 2 provides a concise overview of the NIR spectrometer employed in the experiments, delves into the theoretical underpinnings of the equipment effectiveness investigation and outlines the structure of the proposed estimation models, which are based on FCMs. In the Section 3, a brief description of the NIR spectra samples is presented. Section 4 presents the experimental results and summarizes the key findings and contributions of this study. Finally, the Appendix A section contains additional investigation results, while Appendix B presents a set of NIR spectra, as measured from a set of indicative wheat samples.

## 2. Materials and Methods

### 2.1. Low-Cost NIR Spectrometers

**Figure 1:**
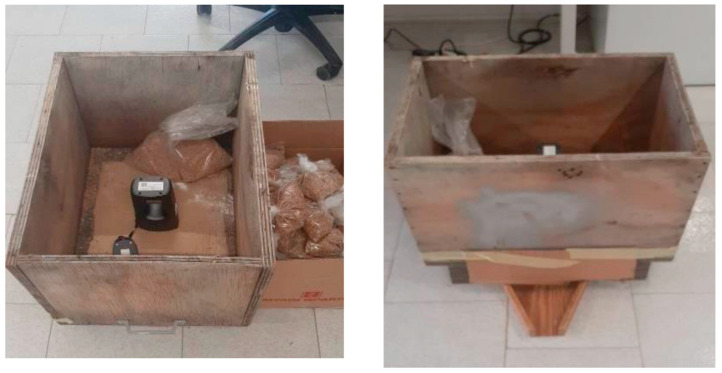
Capturing the spectral profiles of wheat samples within the experimental setup.

The NeoSpectra scanner, designed by Si-Ware, represents a significant advancement in the field of NIR spectroscopy technology. Its compact and portable design makes it a versatile tool for various industries, including agriculture, food processing and pharmaceuticals. One of its standout features is its ability to provide rapid and accurate analysis of material composition in real-time, without the need for sample preparation or complex calibration processes. The scanner’s user-friendly interface and wireless connectivity allow for easy integration into existing workflows, thus enhancing efficiency and quality control. While its affordability and accessibility have broadened the reach of NIR technology, its performance and reliability remain noteworthy, making the NeoSpectra scanner an asset for businesses aiming to streamline their analytical processes and ensure product consistency. The NeoSpectra scanner enables Fourier-Transform Infrared (FT-IR) spectroscopy [16], which operates on the principle of molecular interaction with infrared (IR) light. In this technique, an IR light source emits a broad spectrum of IR radiation, which is directed through a sample. Molecules within the sample absorb specific frequencies of IR light, causing them to vibrate and undergo changes in their molecular energy states. The transmitted or reflected light is then collected and subjected to an interferometer, which modulates the IR beam. The resulting interferogram is transformed using the Fast Fourier Transform (FFT) algorithm, producing an IR spectrum that represents the intensity of the absorbed IR light as a function of frequency. Therefore, in the present study, the specific equipment is investigated in a controllable environment. The experimental setup to capture the NIR spectrums of the studied wheat and flour samples is presented in Figure 1.

### 2.2. Investigation on the Effectiveness of Low-Cost Spectrometer NeoSpectra Scanner

**Figure 2:**
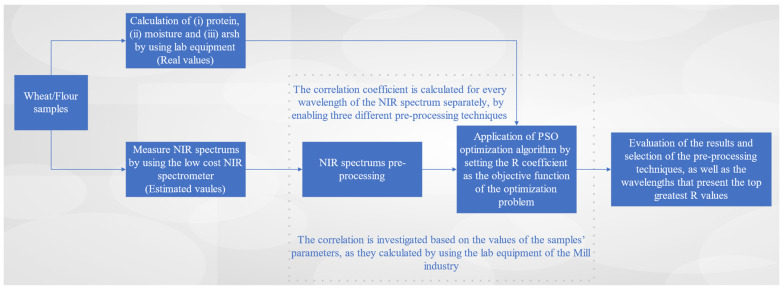
Exploration process of the correlation between the NIR spectra and estimation parameters.

Estimation models play a pivotal role in data analysis and decision-making across various fields. These models are designed to predict or estimate unknown values based on the available data and mathematical algorithms. They are invaluable tools in fields such as the food industry, enabling us to make informed decisions and draw meaningful conclusions from data. Estimation models can range from simple linear regressions to complex machine learning algorithms, depending on the complexity of the problem at hand. Many estimation models in the literature are based on NIR spectrum analysis. NIR spectroscopy involves measuring the interaction of NIR light with a sample, resulting in a spectrum that contains valuable information about the sample’s chemical composition. Estimation models for NIR spectrums are tailored to extract specific parameters or properties from these spectra, such as the moisture content, protein levels or chemical compositions. A typical NIR spectrum is a complex and rich source of information that arises from the interaction of NIR light with matter. Its complexity is a result of the multitude of molecular vibrations, rotations and other interactions that occur within a sample when exposed to NIR radiation. However, only a part of the NIR spectrum includes useful information about the parameters to be estimated. Therefore, the study initially investigates the NIR wavelengths correlated with the parameters to be estimated. In this specific context, the research focuses on five parameters: (i) the protein content of wheat seeds; (ii) the moisture content of wheat seeds; (iii) the protein content of flour samples; (iv) the moisture content of flour samples; (v) the ash content of flour samples. Figure 2 illustrates the assessment of the NIR spectral wavelengths’ effectiveness in estimating these parameters. For each parameter, an estimation model is designed.

**Figure 3:**
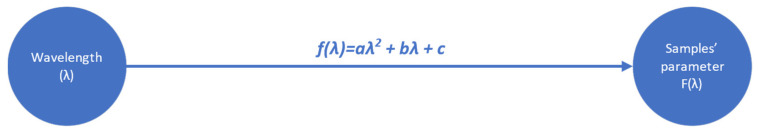
Assumed relation between the wavelengths of the NIR spectra and the samples’ parameters to be estimated.

The process of Figure 2 is employed to identify specific wavelengths within the spectra obtained from the experimental procedure, including pre-processed spectra. This identification aims to pinpoint the wavelengths that exhibit the strongest correlations with the variables that the system under design seeks to estimate. Unlike many correlation models that rely on linear regression, our approach in this study assumes the existence of a polynomial relationship between the desired parameters and the pre-processed spectra. This assumed relationship is depicted in Figure 3.

Where *λ* expresses the wavelength of the NIR spectrum, while *a*, *b* and *c* are coefficients of the second order polynomial and *F*(*λ*) defines the value of the parameter to be examined.

Regarding the investigation into the individual correlations of wavelengths, this research entails assessing their relationships both with the spectra obtained and with the application of the aforementioned preprocessing techniques. The primary focus is on optimizing the correlation coefficient, which serves as the objective function. This optimization aims to align the model for estimating the desired parameter with the data generated by the existing industrial equipment. 

Hence, the optimization challenge at hand involves determining the coefficients (*a*, *b* and *c*) depicted in Figure 2 to maximize the correlation coefficient (*R*). Following this rationale, it is anticipated that wavelengths devoid of any pertinent information about the desired parameter will yield a low maximum correlation coefficient, whereas wavelengths containing relevant information will exhibit a high degree of correlation. The correlation coefficient is formally defined by the following equation (Equation (1)):

(1) $$R_{x,y}=\frac{\sum_{i=1}^{n}\left[{(x}_{i}-\bar{x}){(y}_{i}-\bar{y})\right]}{\sqrt{\sum_{i=1}^{n}\left[{{(x}_{i}-\bar{x})}^{2}{{(y}_{i}-\bar{y})}^{2}\right]}}$$

where *x* and *y* represent the two sets of variables for which the degree of correlation between them is examined.

This study aims to identify the NIR wavelengths that are most significantly influenced by the values of the parameters under investigation. Utilizing the findings of this investigation, we will select the top five wavelengths with the highest correlation coefficients. These selected wavelengths will serve as the foundation for designing an estimation model based on the principles of FCMs theory.

### 2.3. NIR Spectrums Pre-Processing

One of the challenges of NIR spectroscopy is the sensitivity that it presents to the environmental conditions. The existence of light noise significantly affects the accuracy of the measurements [17]; it arises from various sources, including electronic fluctuations, environmental factors and imperfections in the measurement setup. Noise can undermine the quality of NIR spectra, leading to inaccurate interpretations and reduced reliability of analytical results. Effective noise management is crucial for extracting meaningful information from the spectra. Different techniques have been employed in order to mitigate noise and enhance measurement clarity. In the present study, three different pre-processing techniques are enabled: (i) the multiplicative scatter correction; (ii) the first derivatives; (iii) the Savitzky–Golay filters.

#### 2.3.1. Multiplicative Scatter Correction

**Figure 4:**
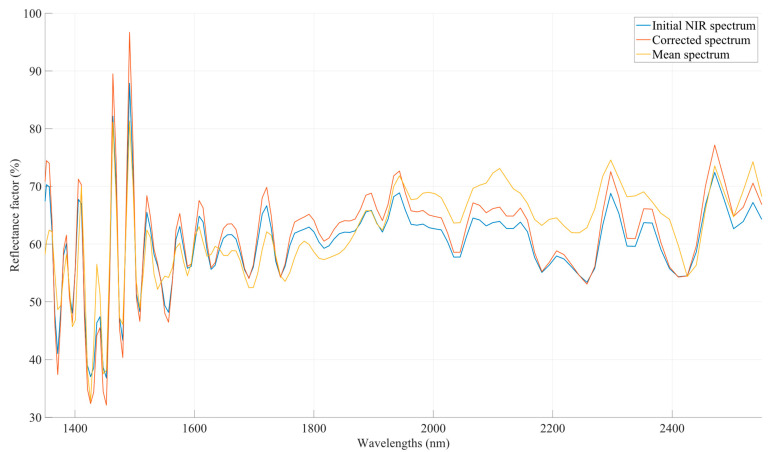
A preprocessed NIR signal after the application of MSC transformation. The spectra have been corrected. The background spectrum (reference signal) has been subtracted in order to isolate the true sample signals.

The established theoretical framework for the study of light behavior states that when light undergoes diffusion after reflection, it induces scattering, which has a multiplicative effect on spectra. Consequently, these spectra become contingent on both scattering and the chemical composition of the reflective material. Given the substantial impact of scattering on spectral behavior, it becomes imperative to apply a method capable of disentangling its influence from the spectra [18]. As such, in line with the literature [19], the most prevalent technique for spectral correction is the Multiplicative Scatter Correction (MSC). In this method, each spectrum within a dataset is adjusted through rotation and shifting to align as closely as possible with the mean spectrum—a process facilitated by the least squares method. The MSC transformation is mathematically described by the following equation:

(2) $$x_{ik,new}=\frac{\left|x_{ik,old}-a_{i}\right|}{b_{i}}$$

where $x_{ik,old}$ represents the initial intensity value of NIR light reflection for sample *i* at wavelength *k* before the MSC transformation. After this transformation, $x_{ik,new}$ represents the updated value. Here, $a_{i}$ signifies the estimated impact of specular reflection on the acquired spectrum, and (1/$b_{i}$) denotes the estimated influence of scattering on the spectrum. It is important to note that these constants, $a_{i}$ and $b_{i}$, are determined through the application of least squares regression. This statistical method correlates each spectrum with the mean spectrum derived from all the spectra considered in the transformation. Equation (2) stems from the spectrum model for sample *i*, which conforms to Equation (3) for each wavelength k:

(3) $$x_{ik}=a_{i}+b_{i}\bar{x}+e_{ik}$$

where $e_{ik}$, is the model error corresponding to the phenomena that distort the useful information of the spectrum and which cannot be modeled using any additional or multiplicative term. Figure 4 depicts the transformation of a NIR signal using the MSC.

#### 2.3.2. First Derivatives

**Figure 5:**
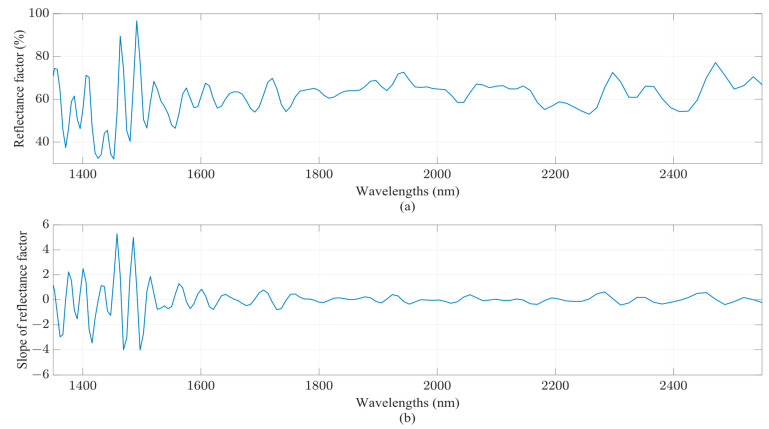
NIR spectrum of a wheat sample, captured using the NeoSpectra scanner, and its first derivatives’ signal: (**a**) the reflectance factor of the NIR spectrum; (**b**) the slope of the reflectance factor of the NIR spectrum. The spectra have been corrected. The background spectrum (reference signal) has been subtracted in order to isolate the true sample signals.

The first derivative indicates the slope of a curve at any given point of it. Its use in NIR signals is important as it removes the baseline from the spectra. The use of first derivatives in a system for calculating chemometric parameters contributes decisively to the removal of unwanted effects in the background, as well as to the removal of noise, resulting in improved spectra for analysis [20,21]. A good approach for calculating derivatives in discrete signals is considered to be the calculation of the difference between two consecutive points and their distance. Therefore, the first derivatives, within the framework of their use in the system under design, are calculated from the following equation:

(4) $$x^{i}=\frac{x_{n}-x_{n-1}}{h}$$

where *x* is the value of a given spectrum for the wavelengths *n* and *n* – 1, and *h* is the distance between the two wavelengths. In Figure 5, an example of a spectrum is depicted, as well as the calculation of its first derivatives.

#### 2.3.3. Smooth Filtering

**Figure 6:**
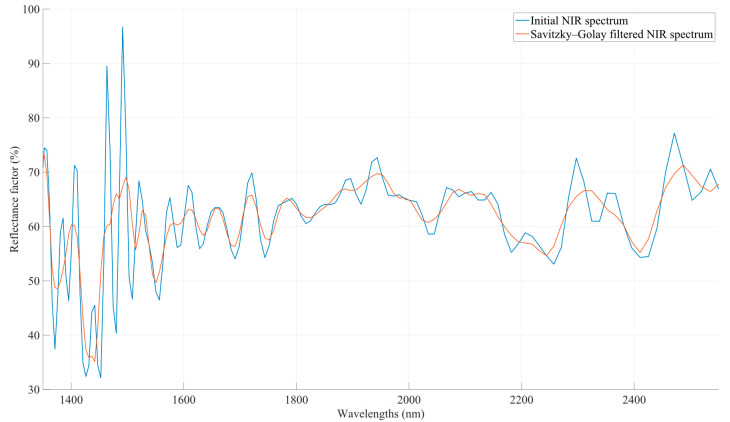
Smoothing filter to a first derivatives’ signal of a NIR spectrum. The spectra have been corrected. The background spectrum (reference signal) has been subtracted in order to isolate the true sample signals.

Savitzky–Golay filters are a valuable tool in the realm of NIR spectroscopy preprocessing. These filters are used to smooth spectral data while preserving the spectral features, making them especially useful for reducing noise and enhancing signal clarity. They work by fitting a polynomial to a small window of data points and then estimating the value at the center of the window. This process is applied iteratively across the entire spectral dataset. In NIR spectroscopy, where precise spectral information is crucial for accurate analysis, Savitzky–Golay filters can effectively reduce the noise caused by random fluctuations and measurement artifacts. By employing these filters during preprocessing, researchers can enhance the quality of NIR spectra, leading to more accurate and reliable analytical results in applications such as chemical analysis, material identification and quality control in various industries [22]. The basic equation of the Savitzky–Golay filter is presented in Equation (5):

(5) $$\hat{y_{k}}=\frac{1}{2s+1}\sum_{m=-s}^{s}c_{m}y_{k+m}$$

where $c_{m}$ is the *m*-th coefficient of the Savitzky–Golay filter, determined based on the degree of the polynomial and the size of the window, $y_{k+m}$ is the original data points within the window, with *m* varying between −*s* and *s*, and $\hat{y_{k}}$ is the smoothed value at the wavelength *k*. Figure 6 illustrates an example of the application of the Savitzky–Golay filter to a typical NIR signal.

### 2.4. Fuzzy Cognitive Maps and Design of the Parameter Estimation Models

**Figure 7:**
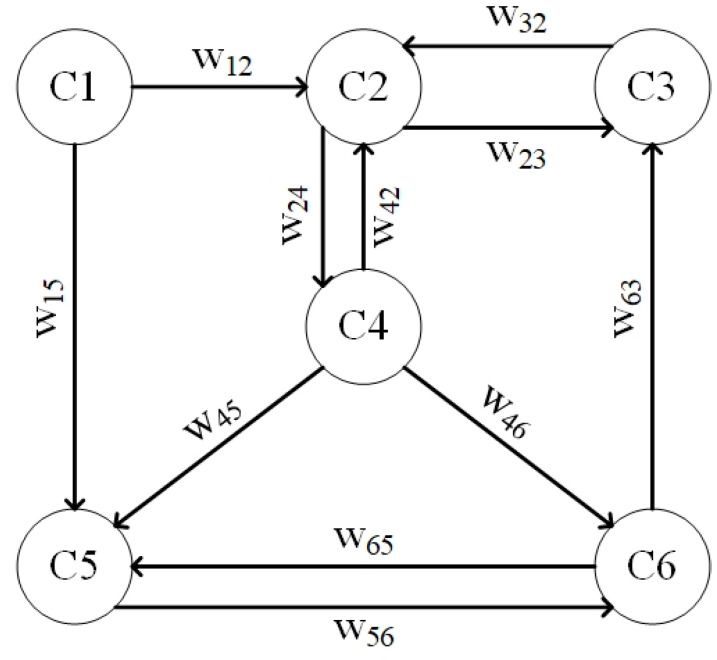
Structure of a typical FCM. *C_i_* defines the value of the node *i*, and *w_ij_* defines the effectiveness of node *i* to node *j* (weight). The value of each node is calculated based on the sigmoid activation function.

In the context of designing the model for estimating the desired parameters in grains and flours, the theory of FCMs has been applied. This selection is underpinned by the intrinsic benefits offered by FCMs, including their abstract framework, inherent flexibility and capacity to adapt to dynamic variations that might arise among interrelated parameters. FCMs, introduced by Kosko [23], present a structured approach to grappling with intricate management and control challenges within complex, nonlinear systems characterized by uncertainties [24]. The guiding principles of FCMs are rooted in symbolically depicting and elucidating the multifaceted phenomena governing such intricate systems, effectively representing logical interconnections between these phenomena [24]. Consequently, FCMs comprise nodes that encapsulate system characteristics and connections that delineate the magnitude and the way one characteristic influences another, achieved through the utilization of weighted interactions. A representative configuration of a typical FCM is illustrated in Figure 7.

The weights in an FCM can be (i) positive, if there is a positive correlation between two nodes (*w_ij_* > 0); (ii) negative, if there is a negative correlation between two nodes (*w_ij_* < 0); (iii) zero, if there is no correlation between the nodes (*w_ij_* = 0). Therefore, the correlations between the different nodes of a typical FCM can be described by the weight matrix, as presented in Equation (6):

(6) $$W=\left[\begin{array}{cccccc} 0 & w_{12} & 0 & 0 & w_{15} & 0\\ 0 & 0 & w_{23} & w_{24} & 0 & 0\\ 0 & w_{32} & 0 & 0 & 0 & 0\\ 0 & w_{42} & 0 & 0 & w_{45} & w_{46}\\ 0 & 0 & 0 & 0 & 0 & w_{56}\\ 0 & 0 & w_{63} & 0 & w_{65} & 0 \end{array}\right]$$

Based on the weights that are defined between the nodes, the values of these can be calculated according to Equation (7):

(7) $$A_{i}(k+1)=f(A_{i}\left(k\right)+\sum_{\begin{array}{c} j=1\\ j\neq i \end{array}}^{n}w_{ji}A_{j}(k))$$

where *A_i_* is the value of node *i*, *Aj* is the value of the nodes that are correlated with node *i*, while the parameter *k* denotes the number of iterations that are performed, until the *A_i_* converges to a value [25,26]. The activation function *f* defines the range of values in which the value of node *i* varies. According to the literature, the most common activation function is the sigmoid function, as defined in Equation (8):

(8) $$f(x)=\frac{1}{1+e^{-\lambda x}}$$

**Figure 8:**
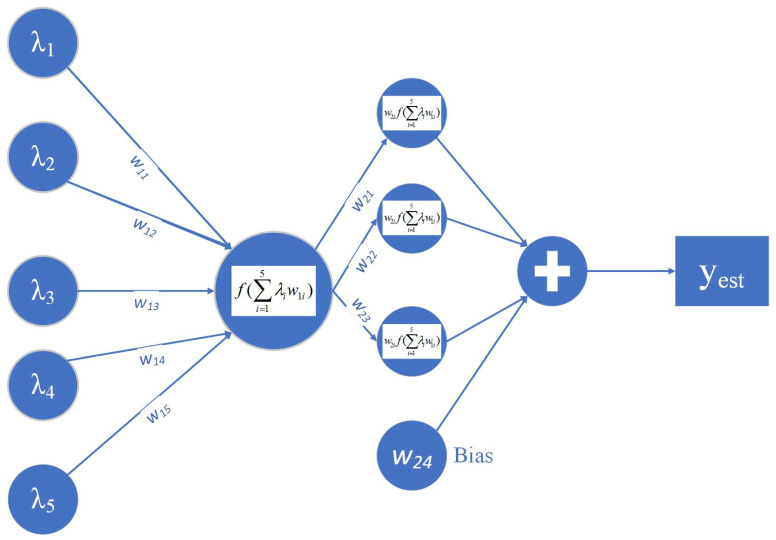
FCM framework for estimating wheat and flour parameters. The nodes *λ_i_* of the FCM define the reflectance factor of the five wavelengths with the highest correlation to the examined parameter.

Observing the structure and operation of FCMs, it appears that they are suitable for the study of the correlation between the desired parameters in grains and flours and the wavelengths collected using the spectrometer as they have been used in various complex problems with great success [27]. According to the structure of a typical FCM, the problem is reduced to find the appropriate weights, so that the proposed model can estimate the desired parameters with great accuracy. In this context, the research team uses the Particle Swarm Optimization (PSO) methodology. The structure of the FCM, which is applied for the estimation of the required parameters (protein, moisture and ash), is presented in Figure 8.

Based on the structure of the parameter estimation system, as presented in the above figure, the problem is reduced to find the weights of the FCM, so that it calculates the desired parameters—whether it is protein, moisture or ash—minimizing the root mean square error. In this context, for a given set of spectra for which the parameter values are known, and based on the research that was conducted in terms of the contribution of the preprocessing methods to the accuracy of parameter estimation, the parameter estimation is reduced to a topic of optimization, in which the estimation system is known as calculating the desired parameters with the minimum error. Therefore, the Root Mean Square Error (*RMSE*) is defined as the objective function of the optimization problem (Equation (9)).

(9) $$RMSE=\sqrt{\frac{1}{n}\sum_{i=1}^{n}{{(Y}_{i}-\bar{Y})}^{2}}$$

## 3. Experimental Setup and Samples Preparation

### Samples Preparation

**Figure 9:**
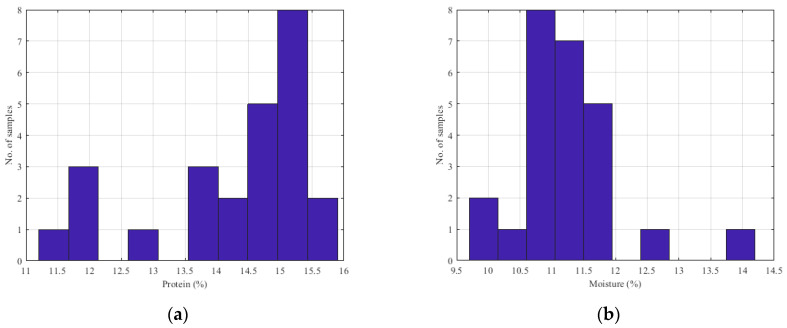
Histograms of the wheat samples’ parameters: (**a**) protein content; (**b**) moisture content.

**Figure 10:**
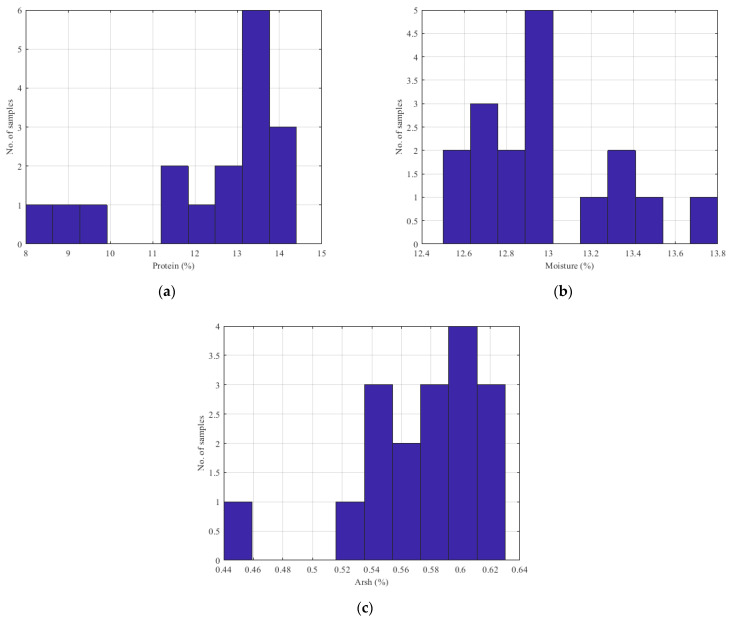
Histograms of the flour samples’ parameters: (**a**) protein content; (**b**) moisture content; (**c**) ash content.

The data used to investigate the effectiveness of the spectrometer, as well as to design the parameter estimation model, came from an experimental process that took place in the industry. Specifically, 25 samples of wheat and 17 samples of flour were examined. For each sample, 10 different NIR spectrums were collected. The exposure time of each measurement was set to 5 s. For these samples, the analysis was performed in the industry’s chemistry laboratory in order to determine the protein, moisture and ash contents (ash was measured for flour). The histograms of the collected wheat samples are presented in Figure 9. Figure 10 provides the histograms of the parameters of the flour samples. The mean value and the variance of the gathered samples, as the standard deviation (SD) and the standard error (SE), are presented in Table 1. Based on the histograms depicted in Figure 9 and Figure 10, along with the average and variance values, it is evident that the samples under examination do not exhibit a uniform distribution. This non-uniformity can be attributed to the fact that these samples were collected from various stages of the production line in the milling industry and the fact that a typical mill flour industry processes a specific kind of seed. Consequently, they represent the diverse types of seeds and flours processed by the industry. In order to calculate the reference values of the studied wheat and flour samples, a dedicated high-accuracy NIR analyzer was used. The experienced chemicals of the mill industry calculate the reference values, based on the analyzer.

## 4. Results Evaluation and Discussion

### 4.1. Results on the Investigation of Wavelengths’ Effectiveness on Wheat and Flour Chemical Parameters

The analysis of the effect of each wavelength in the NIR spectrum on its correlation with the examined parameter was conducted through the investigation of various study cases. In each study case, a combination of the aforementioned preprocessing methods was applied, followed by an examination of the correlation exhibited by each wavelength of the preprocessed spectra with the desired parameters to be estimated. The aforementioned evaluation was applied to samples of cereals. The results of this assessment are presented in detail in Appendix A, illustrating both the correlation of each NIR wavelength with the corresponding parameter (wheat protein and moisture) and the histogram of the correlation coefficients for each wavelength. It is observed that during the protein examination, where all three preprocessing methods were applied and the correlation between each wavelength and the desired parameter was subsequently examined, most wavelengths in the NIR spectrum displayed a high percentage of correlations compared to the other combinations of the pre-processing techniques. Regarding the application of the smoothing filter, various windows were examined. The windows that appeared to contribute to the increased correlation coefficient between the wavelengths and the desired parameters were those with lengths of 9 and 13. Consequently, in the preprocessing methods applied to the NIR spectra for the estimation models of protein in wheats, the study case containing all three preprocessing methods was selected. In this instance, wavelengths were found that exhibited correlation values of up to 0.7. Regarding the smoothing filter window, a window size of 9 was chosen as, according to the figures in Appendix A, wavelengths with correlation coefficients up to 0.7 were observed. Concerning the selection of wavelengths to be used as inputs in the FCM, the wavelengths that exhibited the five highest correlations compared to the others were selected. These wavelengths demonstrated correlations ranging between 0.6 and 0.7. In contrast, regarding the moisture content, it was concluded that the application of the smooth filter degrades the correlation between the wavelengths and the moisture parameter. The results indicate that only the application of the MSC transformation can achieve the most wavelengths with a correlation coefficient above 0.7. Finally, from the present study, it is extracted that most of the wavelengths of the NIR spectrum do not present a correlation with the protein and moisture of the samples when they are examined separately. However, this fact does not lead to the conclusion that they cannot contribute to the estimation of the required parameters. The present fact needs another form of investigation by investigating the different features that are extracted by the whole spectra. In the design of our estimation models, we employed a specific strategy tailored to the nature of the targeted variables. For the protein estimation model, we utilized all three pre-processing techniques to extract the five values that demonstrate the highest correlations with the wavelengths and protein content. Conversely, for the moisture estimation model, we exclusively applied the MSC transformation, enabling us to selectively identify the five wavelengths that exhibit the strongest correlations with moisture content.

### 4.2. Optimization Results of the FCMs Estimation Models

In Table 2 and Table 3, the weightings of the FCMs for the estimation of the wheat and the flour parameters, as depicted in Figure 8, are presented. This graph serves as the foundation for our estimation model for each parameter for the wheat and flour samples. While the FCM’s structure remains consistent across all of the estimation models, the weight within the graph undergoes variations. Upon analyzing these weights, it becomes evident that the wavelengths with the most significant influence on estimating both the protein and moisture content in cereals are those situated towards the higher end of the NIR spectrum (with values exceeding 2000 nm). However, it is worth noting that specific wavelengths scattered throughout the NIR spectrum exhibit sensitivity to the parameters of interest. As wavelengths approach the visible spectrum, their impact on the parameter estimation diminishes. Remarkably, the wavelengths displaying the strongest correlation with the moisture estimation fall within the range of 1728 to 2488 nm. In contrast, the protein estimation model highlights wavelengths in the range of 1375 to 2440 nm. A notable trend emerges as most of the wavelengths positively contribute to the estimation of both parameters, evident by their positive weights. Essentially, this implies that as the NIR light reflectance value from cereals increases, so does the estimated protein content. In both models, there is only one wavelength displaying a negative correlation.

As FCMs are being introduced for the first time in the design of parameter estimation models for cereals and flours, based on NIR spectra, their effectiveness in the parameters under examination is investigated, by comparing their efficiency with the application of the PLS method. Therefore, in parallel with the FCMs, the PLS regression method is also used, on the same spectra. This specific method was selected over other classical methods, as it has been applied to the same problem with satisfactory results. It presents a robust methodology for addressing multicollinearity and managing high-dimensional datasets. Its capacity to efficiently reduce dimensionality not only guards against overfitting but also simplifies the modeling process. Additionally, PLS’s resilience when confronted with limited sample sizes.

Moreover, to validate the FCM models, as well as the optimization method of their weights, the cross-validation technique was used. By repeating the optimization procedure of each FCM graph for a specified number of iterations, the root mean square error was calculated for both the training and testing samples. As the number of the dataset is relatively small, the number of the folds was set to three.

**Figure 11:**
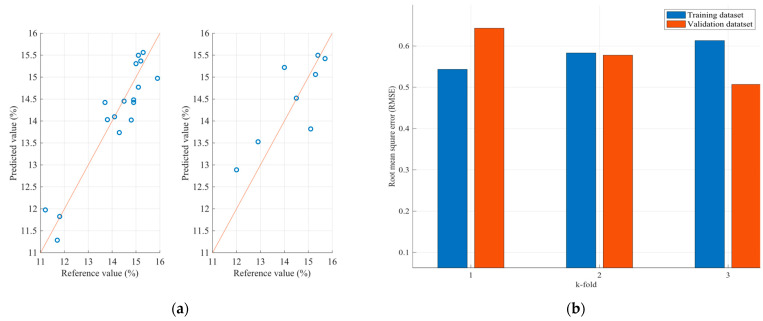

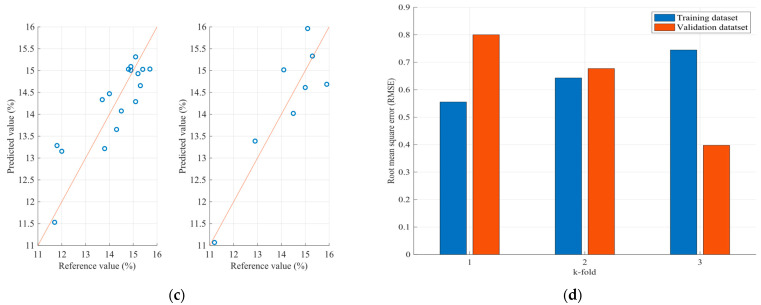
Analysis of the FCM protein estimation model applied to the wheat dataset: (**a**) Regression curves of the FCM model (left curve referring to training dataset and the right to the testing dataset); (**b**) RMSEs as calculated using the k-fold cross validation method for the FCM model; (**c**) Regression curves of the PLS model (left curve referring to training dataset and the right to the testing dataset); (**d**) RMSEs as calculated using the k-fold cross validation method for the PLS model.

**Figure 12:**
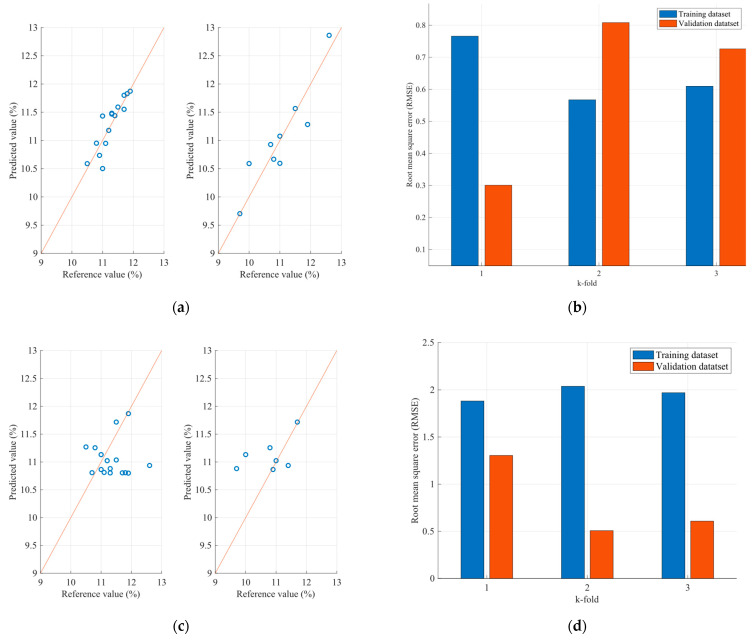
Analysis of the FCM moisture estimation model applied to the wheat dataset: (**a**) Regression curves of the FCM model (left curve referring to training dataset and the right to the testing dataset); (**b**) RMSEs as calculated using the k-fold cross validation method for the FCM model; (**c**) Regression curves of the PLS model (left curve referring to training dataset and the right to the testing dataset); (**d**) RMSEs as calculated using the k-fold cross validation method for the PLS model.

**Figure 13:**
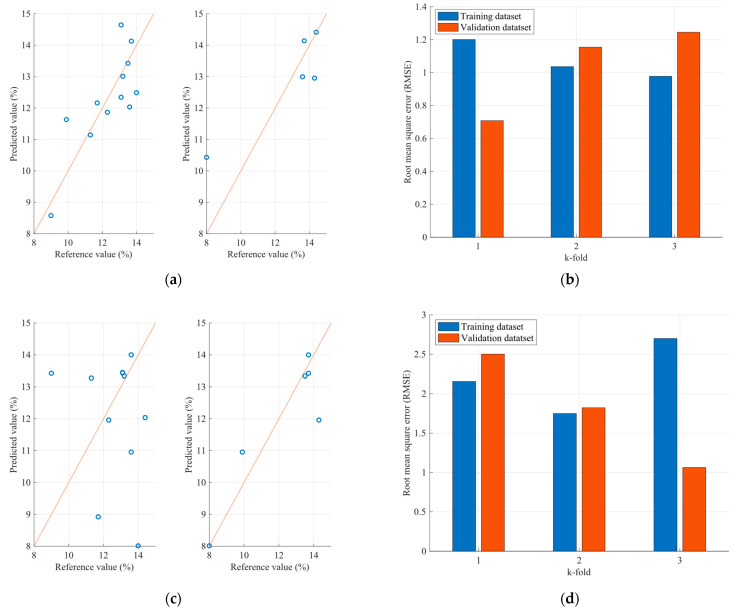
Analysis of the FCM protein estimation model applied to the flour dataset: (**a**) Regression curves of the FCM model (left curve referring to training dataset and the right to the testing dataset); (**b**) RMSEs as calculated using the k-fold cross validation method for the FCM model; (**c**) Regression curves of the PLS model (left curve referring to training dataset and the right to the testing dataset); (**d**) RMSEs as calculated using the k-fold cross validation method for the PLS model.

**Figure 14:**
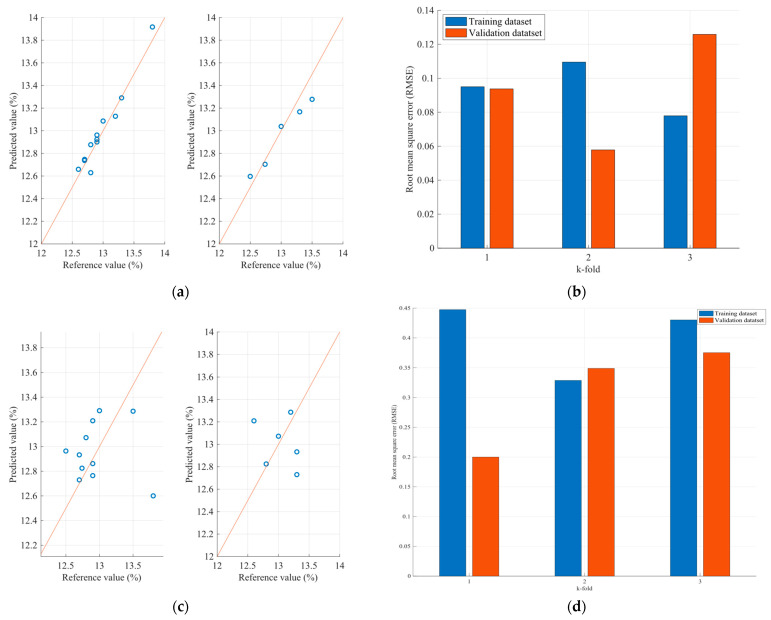
Analysis of the FCM moisture estimation model applied to the flour dataset: (**a**) Regression curves of the FCM model (left curve referring to training dataset and the right to the testing dataset); (**b**) RMSEs as calculated using the k-fold cross validation method for the FCM model; (**c**) Regression curves of the PLS model (left curve referring to training dataset and the right to the testing dataset); (**d**) RMSEs as calculated using the k-fold cross validation method for the PLS model.

**Figure 15:**
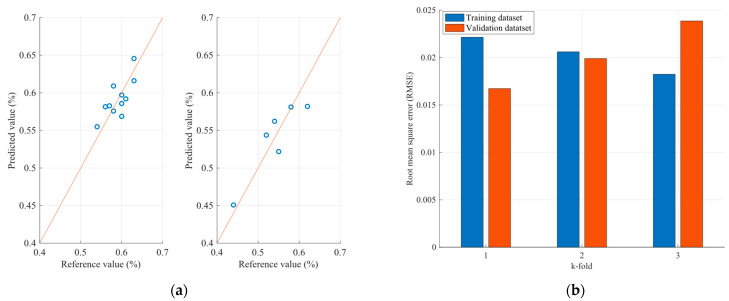

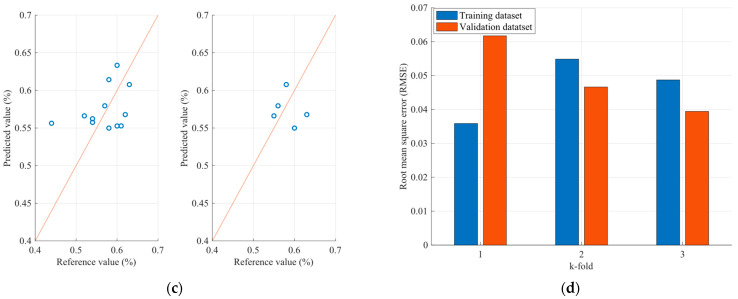
Analysis of the FCM ash estimation model applied to the flour dataset: (**a**) Regression curves of the FCM model (left curve referring to training dataset and the right to the testing dataset); (**b**) RMSEs as calculated using the k-fold cross validation method for the FCM model; (**c**) Regression curves of the PLS model (left curve referring to training dataset and the right to the testing dataset); (**d**) RMSEs as calculated using the k-fold cross validation method for the PLS model.

Therefore, Figure 11 and Figure 12 present the protein and moisture results regarding the wheat samples accordingly, while Figure 13, Figure 14 and Figure 15 present the results regarding the protein, moisture and ash parameters. Figure 11a, Figure 12a, Figure 13a, Figure 14a and Figure 15a present the regression curves of the training and testing datasets using the FCM models, Figure 11b, Figure 12b, Figure 13b, Figure 14b and Figure 15b depict the k-fold RMSEs using the FCM models, while Figure 11c, Figure 12c, Figure 13c, Figure 14c and Figure 15c and Figure 11d, Figure 12d, Figure 13d, Figure 14d and Figure 15d present the regression curves and the k-fold RMSEs using the PLS regression methodology.

Regarding the wheat protein and moisture estimation models, both exhibit a commendable performance, boasting correlation coefficients exceeding 0.9. The largest observed discrepancy is merely 1%, which was found within the same sample for both the protein and moisture content. Furthermore, it is noteworthy that the protein estimation model demonstrates a stronger correlation with the moisture model. This conclusion is drawn from the root mean square errors observed in the k-folds of the training process. While the protein estimation model yields nearly 0.6, the moisture model displays root mean square errors ranging over 0.7. Nevertheless, these errors remain relatively minor, supporting the overall reliability of our value estimations. In summary, our study employed a fuzzy cognitive map to estimate the protein and moisture content in wheats. The results affirm the effectiveness of our model, showcasing correlation coefficients exceeding 0.9 for both parameters. The maximum deviations between the estimated and actual values were limited to a mere 1%. Additionally, we observed that certain wavelengths in the NIR spectrum are particularly sensitive to structural changes in cereals that affect the protein and moisture content. While these findings are promising, further validation on a larger dataset is warranted to affirm their general applicability.

On the other hand, the parameter estimation models for flours exhibit a different behavior. Initially, it is observed that—unlike the grain estimation models—in flours, at least two out of five wavelengths that significantly affect the desired variables have a negative correlation with the variables. Additionally, the range of values is smaller, as the wavelengths with the highest correlations range between 1490 nm and 2055 nm. This may be due to the value ranges of the grains processed by the factory and the flours produced, as well as the different textures between the grains and flours, resulting in an influence on the spectra acquisition. The results of the application of the estimation models for the parameters of the flours are presented, as well as the root mean square errors for each of the three parameters. Observing the mean square error that appears in the case of protein, it seems that specific samples exhibit relatively large errors between the actual and estimated values. It is estimated that one of the reasons for this anomaly is due to the dust generated by the flour in the environment where the NIR spectra are taken, resulting in additional noise and, therefore, some samples showing significant deviations. Furthermore, larger errors are observed in the case of the flour samples compared to the grain samples. The above can be considered as a stimulus for further studies on this specific issue to improve the ability to approximate the desired parameters for both flours and grains. However, the results observed in the case of moisture and ash appear to be better, as according to the regression curves, most of the samples in both the training and the test datasets appear to be closer to the regression line. 

Comparing the regression curves and the RMSEs between the FCM models and the corresponding PLS models for each desired parameter, it is observed that, in all cases, the results of the FCM models are better than those of the PLS. In some cases, such as in the case of moisture estimation in cereals and flours, there is a significant reduction in errors when FCM models are used. This is also extracted from Table 4, which for each type of sample and for each parameter, presents the mean RMSE of the k-fold cross validation method for both the FCM models and the PLS models.

In all of the FCMs that were designed for the estimation of parameters in cereals and flours, it is observed that the models based on FCMs are more effective than the estimation models based on the PLS method. This is due to the fact that the FCMs, through their robustness, can approach the dynamic behavior of various mechanisms, such as those studied, at a satisfactory level. However, it should be noted that the number of principal components in the PLS method was selected to be equal to three, which is a small value. This value was selected as the number of samples is relatively small. Therefore, based on the present research, the results that emerge for the inclusion of FCMs in the field of designing parameter estimation models are quite encouraging. However, in order to become established in this field of parameter estimation models, there should be other studies on the integration of FCMs and their use in the design of parameter estimation models. In addition, the choice of features that arise from the NIR spectra and are taken into account in the FCM also plays an important role. Therefore, the choice of other features, such as the energy of the NIR spectrum, as well as other statistical measures, could lead to an increase in their effectiveness.

Therefore, according to the initial analysis of the effectiveness of the proposed models based on the usage of a low-cost NIR spectrometer, the results indicate that by enabling computational intelligence algorithms, the usage of low-cost NIRs can provide useful information regarding the parameter estimation of protein, moisture and ash in mill industry applications. However, there is a need to examine the effectiveness of both models and the low-cost handle NIR in more samples in which the values of the samples’ parameters will be uniformly distributed. This is a challenge as mill flour industries work on specific kinds of wheat seeds and flours and it is difficult to create datasets that allocate the protein and moisture values along a uniform distribution.

## 5. Conclusions

In this study, a cost-effective NIR spectrum was employed to assess its suitability for application within a milling industry process. The objective was to utilize this equipment to estimate key parameters, including the protein and moisture content in wheat seeds, as well as the protein, moisture and ash levels in flour samples. Notably, the approach deviates from conventional state-of-the-art NIR spectrometry, which typically rely on InGaAs sensors, as we harnessed a low-cost Fourier-Transform Infrared spectroscopy technology-based NIR sensor. To enhance the accuracy of our measurements, a novel calibration model is introduced, incorporating a training phase based on the PSO optimization algorithm. This process aimed to fine-tune a specialized FCM model specifically tailored for parameter estimation. The design of the FCM model was based on the wavelengths extracted from the captured NIR spectra as these wavelengths displayed heightened correlations with the parameters under investigation. The evaluation of the low-cost spectrometer, along with the integration of CMOS technology into NIR spectrometry, demonstrates its capability to measure chemical parameters effectively. However, further enhancements in the accuracy can be achieved by employing advanced calibration models that leverage AI techniques. Moreover, expanding the dataset used for model calibration, including the proposed FCM model in this paper, is recommended. Nonetheless, it is imperative to conduct long-term experiments to adequately assess the equipment’s performance over extended durations. Considering the promising outcomes of this study, our future research will focus on integrating low-cost NIR sensors into the mill flour industry, exploring alternative algorithms for calibration, such as reinforcement learning. Moreover, other variables and features from the gathered NIR spectra will be examined, such as statistical parameters, to investigate the potential improvement in the FCMs’ accuracy. Finally, the examined NIR spectrometer will be evaluated and tested on in-line measurements, based on a larger dataset of wheat and flour samples.

## Figures and Tables

**Table 1:** Statistical values of the examined parameters in the wheat and flour.

Sample	Number of Samples	Parameter (%)	Average	Variance	SD	SE
Wheat	25	Protein	14.25	1.77	1.33	0.27
		Moisture	11.30	0.74	0.86	0.17
Flour	17	Protein	12.50	3.62	1.904	0.462
		Moisture	13.00	0.12	0.342	0.082
		Ash	0.58	0.002	0.047	0.012

**Table 2:** Values of the FCM estimation model for the wheat parameters.

Weight	Protein Estimator	Selected Wavelength	Moisture Estimator	Selected Wavelength
W_1,1_	3.11	2440.50	2.43	2487.05
W_1,2_	3.58	2381.07	0.03	2194.07
W_1,3_	0.17	2366.66	0.61	1759.74
W_1,4_	−0.11	1380.79	3.21	1736.30
W_1,5_	0.18	1375.93	−1.52	1728.63
W_2,1_	18.00		22.48	
W_2,2_	10.23		5.24	
W_2,3_	12.95		11.17	
W_2,4_	−6.69		−15.38	

**Table 3:** Values of the FCM estimation model for the flour parameters.

Weight	Protein Estimator	Selected Wavelength	Moisture Estimator	Selected Wavelength (nm)	Ash Estimator	Selected Wavelength (nm)
W_1,1_	8.00	2055.69	15.46	1933.73	−9.87	1775.71
W_1,2_	−4.83	2044.94	−14.67	1924.22	0.01	1759.73
W_1,3_	−7.53	1683.97	−0.16	1457.99	0.03	1751.85
W_1,4_	4.05	1375.93	0.03	1405.59	−0.41	1491.35
W_1,5_	−0.71	1371.10	−0.54	1347.49	5.70	1480.06
W_2,1_	0		0		0	
W_2,2_	0		0		0	
W_2,3_	−0.08		0.05		0.01	
W_2,4_	9.01		12.75		1.97	

**Table 4:** Comparison of root mean square errors between the FCM and the PLS estimation models.

Sample	Parameter (%)	FCM Model RMSE	PLS Model RMSE
Wheat	Protein	0.581	0.65
	Moisture	0.412	1.93
Flour	Protein	1.06	2.40
	Moisture	0.09	0.38
	Ash	0.020	0.055

**Figure A1:**
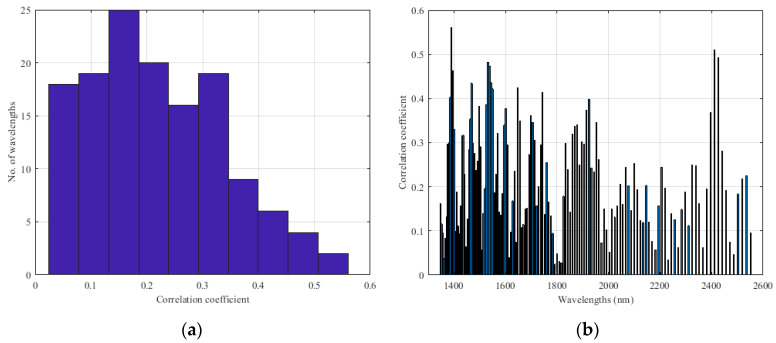
Analysis of wavelength effectiveness in estimating protein content of the wheat samples without applying any preprocessing technique: (**a**) Histogram of the Correlation Coefficients between NIR Wavelengths and Protein Content, (**b**) Correlation Coefficients for Each NIR Wavelength.

**Figure A2:**
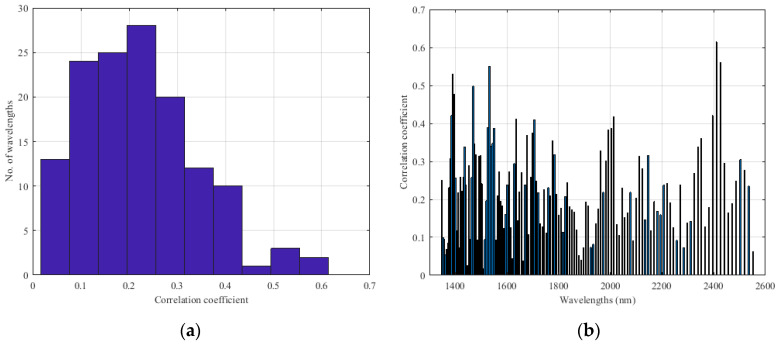
Analysis of wavelength effectiveness in estimating protein content of the wheat samples, by applying the MSC transformation: (**a**) Histogram of the Correlation Coefficients between NIR Wavelengths and Protein Content, (**b**) Correlation Coefficients for Each NIR Wavelength.

**Figure A3:**
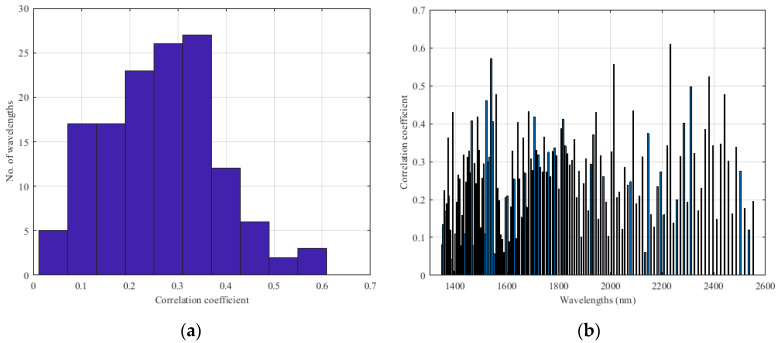
Analysis of wavelength effectiveness in estimating protein content of the wheat samples, by applying the MSC transformation and first-derivatives: (**a**) Histogram of the Correlation Coefficients between NIR Wavelengths and Protein Content, (**b**) Correlation Coefficients for Each NIR Wavelength.

**Figure A4:**
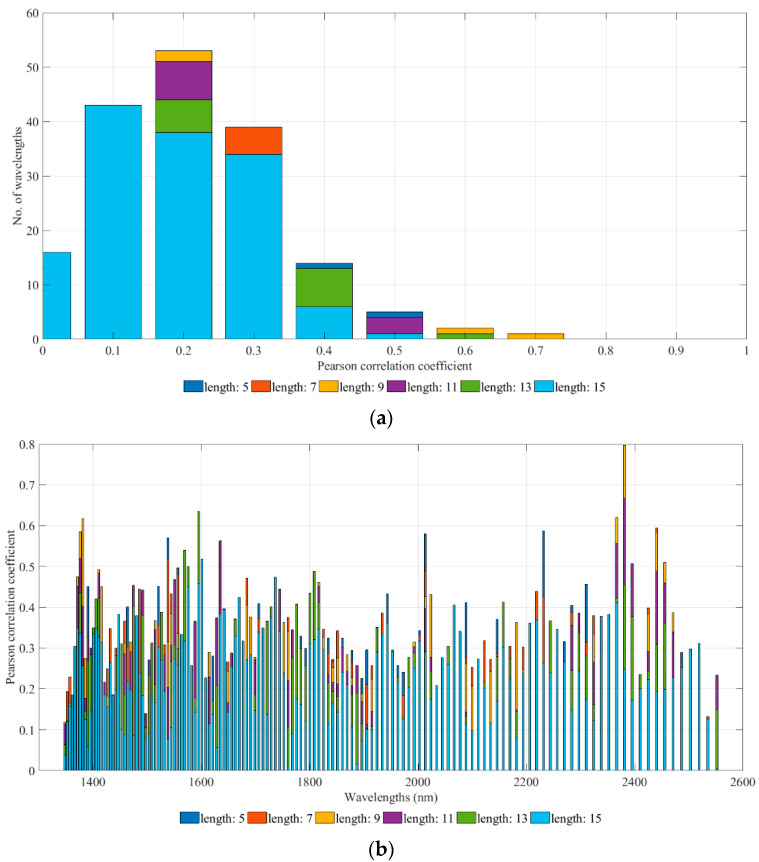
Analysis of wavelength effectiveness in estimating protein content of the wheat samples, by applying the MSC transformation, first-derivatives and smooth filter: (**a**) Histogram of the Correlation Coefficients between NIR Wavelengths and Protein Content, (**b**) Correlation Coefficients for Each NIR Wavelength.

**Figure A5:**
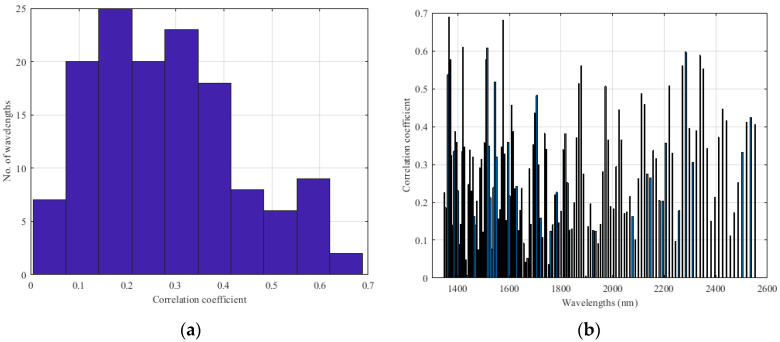
Analysis of wavelength effectiveness in estimating moisture content of the wheat samples without applying any preprocessing technique: (**a**) Histogram of the Correlation Coefficients between NIR Wavelengths and Protein Content, (**b**) Correlation Coefficients for Each NIR Wavelength.

**Figure A6:**
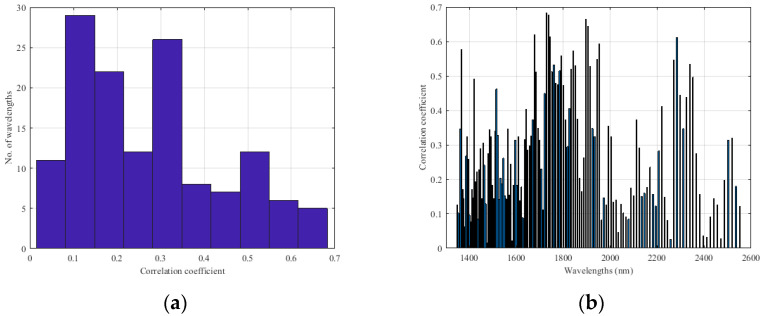
Analysis of wavelength effectiveness in estimating moisture content of the wheat samples, by applying the MSC transformation: (**a**) Histogram of the Correlation Coefficients between NIR Wavelengths and Protein Content, (**b**) Correlation Coefficients for Each NIR Wavelength.

**Figure A7:**
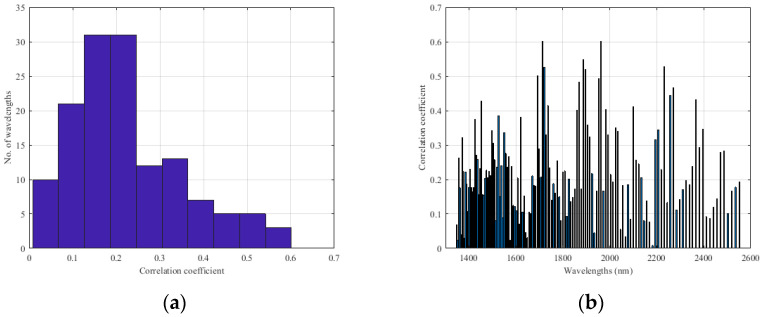
Analysis of wavelength effectiveness in estimating moisture content of the wheat samples, by applying the MSC transformation and first-derivatives: (**a**) Histogram of the Correlation Coefficients between NIR Wavelengths and Protein Content, (**b**) Correlation Coefficients for Each NIR Wavelength.

**Figure A8:**
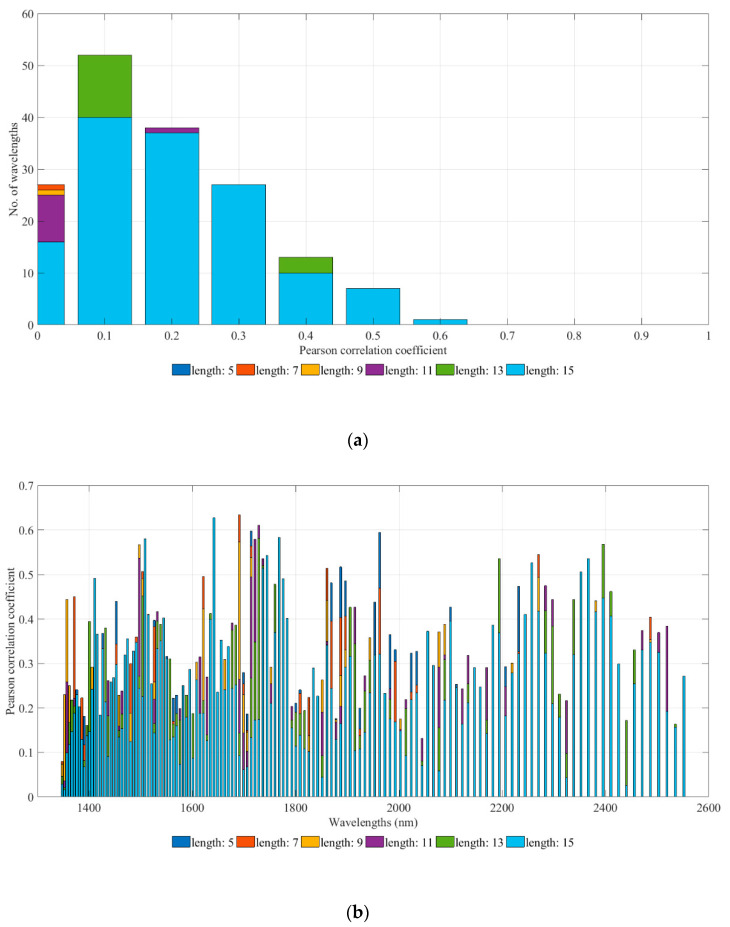
Analysis of wavelength effectiveness in estimating moisture content of the wheat samples, by applying the MSC transformation, first-derivatives and smooth filter: (**a**) Histogram of the Correlation Coefficients between NIR Wavelengths and Protein Content, (**b**) Correlation Coefficients for Each NIR Wavelength.

**Figure A9:**
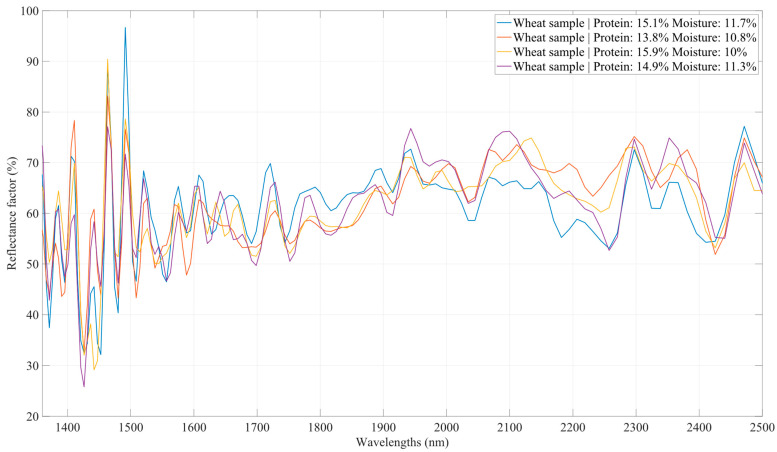
Near-infrared spectra obtained from analysis of four different wheat samples.

## Data Availability

Data are available on request due to privacy restrictions.

## Appendix A

Appendix A showcases the outcomes of a sensitivity analysis conducted using the employed spectrometer, demonstrating the correlation between individual wavelengths and fluctuations in protein and moisture levels within the wheat seeds samples (Figure A1, Figure A2, Figure A3, Figure A4, Figure A5, Figure A6, Figure A7 and Figure A8).

## Appendix B

In Appendix B, Figure A9, NIR spectra are presented from four different cereal samples with varying protein and moisture parameters. The spectra have been corrected. The background spectrum (reference signal) has been subtracted in order to isolate the true sample signals.
